# 1 billion-year-old cell contents preserved in monazite and xenotime

**DOI:** 10.1038/s41598-019-45575-4

**Published:** 2019-06-21

**Authors:** David Wacey, Eva Sirantoine, Martin Saunders, Paul Strother

**Affiliations:** 1 Centre for Microscopy Characterisation and Analysis, The University of Western Australia, 35 Stirling Highway, Perth, WA 6009 Australia; 2 School of Earth Sciences, The University of Western Australia, 35 Stirling Highway, Perth, WA 6009 Australia; 3 School of Molecular Sciences, The University of Western Australia, 35 Stirling Highway, Perth, WA 6009 Australia; 4 Department of Earth and Environmental Sciences, Weston Observatory of Boston College, 381 Concord Road, Weston, MA 02493 USA

**Keywords:** Element cycles, Palaeontology

## Abstract

Exceptional microfossil preservation, whereby sub-cellular details of an organism are conserved, remains extremely rare in the Precambrian rock record. We here report the first occurrence of exceptional cellular preservation by the rare earth element (REE) phosphates monazite and xenotime. This occurs in ~1 billion-year-old lake sediments where REEs were likely concentrated by local erosion and drainage into a closed lacustrine basin. Monazite and xenotime preferentially occur inside planktonic cells where they preserve spheroidal masses of plasmolyzed cell contents, and occasionally also membranous fragments. They have not been observed associated with cell walls or sheaths, which are instead preserved by clay minerals or francolite. REE phosphates are interpreted to be the earliest minerals precipitated in these cells after death, with their loci controlled by the micro-scale availability of inorganic phosphate (P_i_) and REEs, probably sourced from polyphosphate granules within the cells. The strong affinity of REEs for phosphate and the insolubility of these minerals once formed means that REE phosphates have the potential for rapid preservation of cellular morphology after death and durability in the rock record. Hence, authigenic REE phosphates provide a promising new target in the search for the preservation of intra-cellular components of fossilised microorganisms.

## Introduction

**Figure 1:**
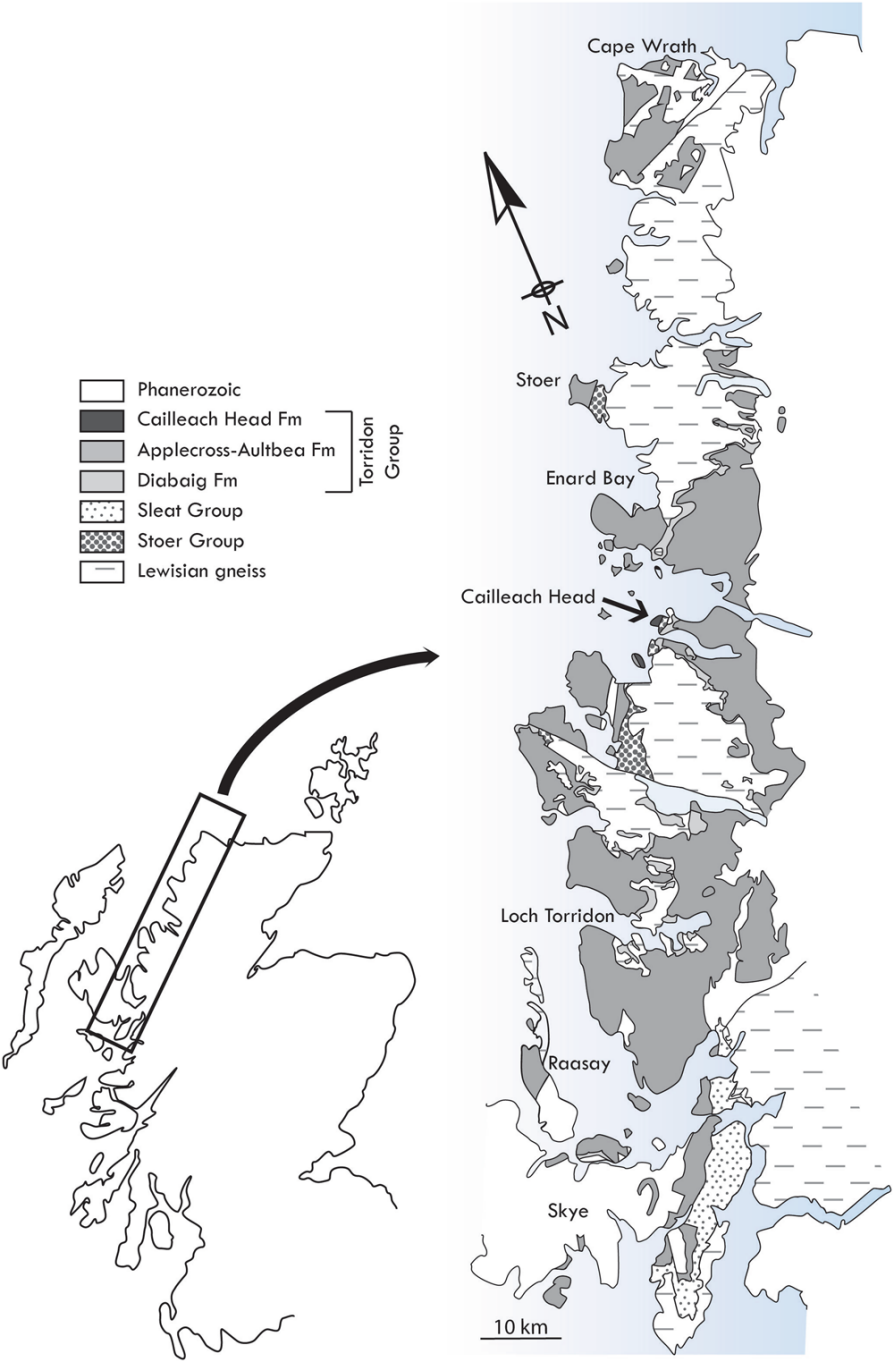
Map of NW Scotland showing generalized bedrock geology, and location of the material sampled for this study from the Cailleach Head Peninsula (small arrow).

The REE phosphates monazite and xenotime are relatively common as minor components of granitic and gneissic rocks, and as detrital grains in sedimentary rocks derived from those sources. However, authigenic sedimentary REE phosphates are rather rare, reported from only a handful of marine and continental sediments as nodules or linings on detrital mineral surfaces^1,2^. The association of REE phosphates with microfossils has, to the best of our knowledge, never been reported. Instead, three-dimensionally preserved cellular remains commonly occur in silica^3^, apatite/francolite^4^, and more rarely pyrite^5^ and clay minerals^6^. We here report authigenic monazite and xenotime occurring within organic-walled microfossils from the Cailleach Head Formation (CHF) of the ~ 1Ga Torridon Group, Northwest Scotland (Fig. 1). In this setting, rapid post-mortem precipitation of these REE phosphates resulted in exceptional preservation of sub-cellular detail in selected cells.

**Figure 2:**
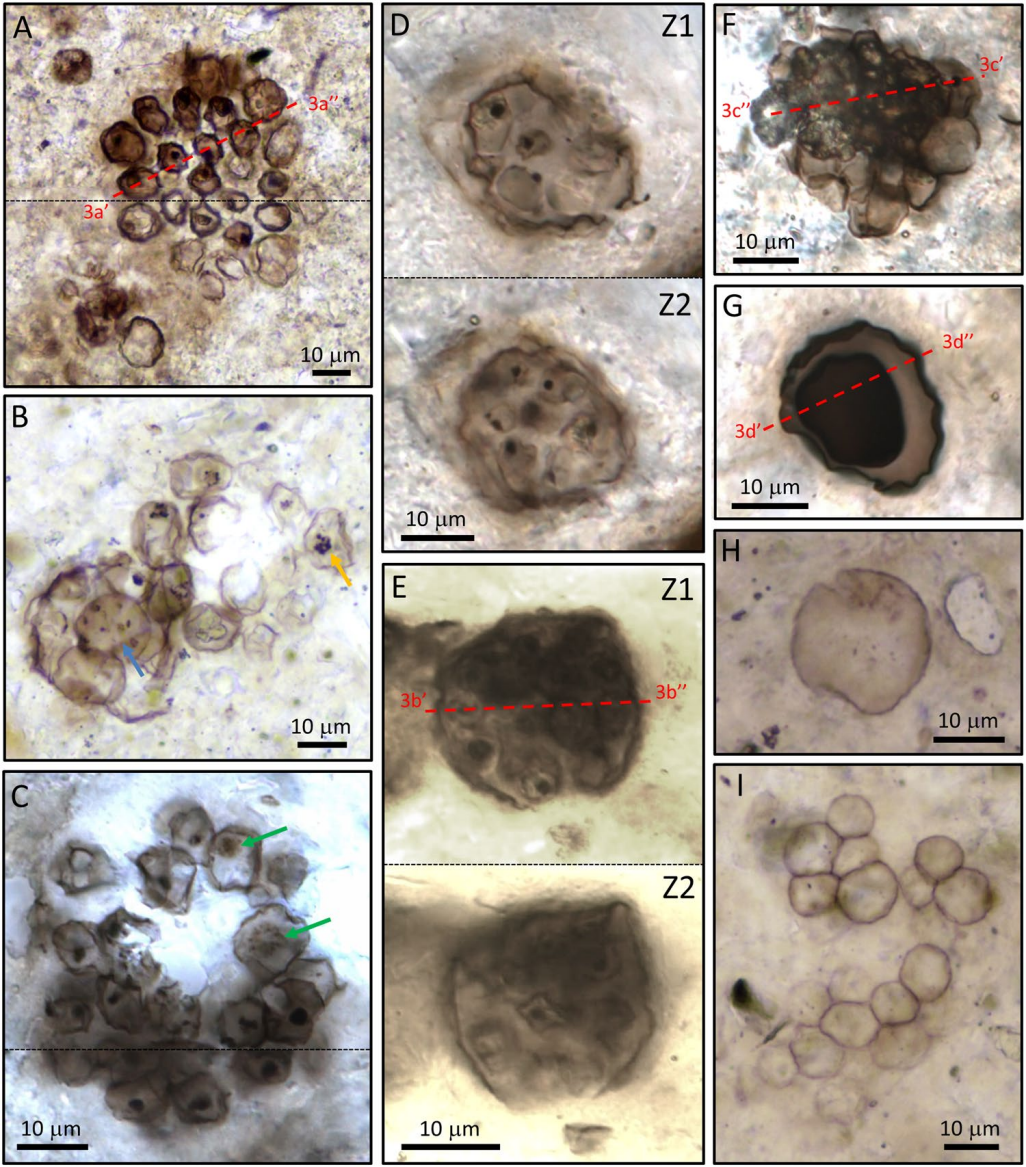
Studied microfossils from the Cailleach Head Formation. (**A**–**C**) Open clusters of cells, probably *Glenobotrydion aenigmatis* Schopf. In (**A**) most cells in the cluster contain single dense, medial, organic intracellular inclusions (ICIs). In (**B**) ICIs are not typically single spots, but instead are dispersed throughout the cell (blue arrow) or occur as smaller internal clusters (orange arrow). In (**C**) the ICIs range from dense condensed spots to larger beaded spheroids (green arrows). (**D**–**E**) Enclosed clusters of small coccoidal cells with ICIs photographed in 2 focal plans, Z1, Z2. Phylogenetic affinity of these is indeterminate. (**F**) Dense cell cluster of unknown affinity with ICIs in a small number of cells. (**G**) Single probably encysted eukaryotic cell of unknown affinity with thick outer (vegetative) cell wall and an inner broadly spherical (cyst?) body. (**H**) Coccoid unicell of unknown affinity possessing a rather thin cell wall. (**I**) Cluster of coccoid cells with mutually adpressed walls that do not contain ICIs. REE phosphates have been observed in the cell types depicted in (**A**–**G**) but not in the cell types depicted in (**H**,**I**).

The CHF comprises the uppermost portion of the Torridon Group, a clastic sequence of sandstones and shales, deposited respectively in fluvial and lacustrine environments^7,8^ that now outcrop over a ~150 km long coastal strip of North-West Scotland, reaching a total thickness of ~6.5 km (Fig. 1). Samples were collected from the Cailleach Head Peninsula, the type locality of the CHF, where the CHF is conformably underlain by the 992 ± 52 Ma Aultbea Formation^9^ and is truncated above by an angular unconformity with Cambrian quartzite of the Eriboll Formation^7^. The CHF contains a series of at least 15 coarsening upwards cyclothems, each comprising two facies that respectively represent deeper water lake deposits and fluvial sediments prograding into a lake^7^, with our study material coming from centimetre-sized phosphatic nodules within the second facies. Microfossils from CHF phosphatic nodules have been described previously^6,10,11^ and consist of a wide array of clusters of coccoids and coccoid unicells, some of which have been interpreted to be of eukaryotic affinity^10^, plus a minor filamentous component. Our study material is dominated by clustered and solitary coccoidal cells (Fig. 2). Clusters of coccoids can occur as loosely arranged groups of ten of more cells with no contact between individual cells (Fig. 2A–C), or as more tightly bound groups surrounded by an external membrane (Fig. 2D–E). Some clusters also contain cells with mutually compressed walls (Fig. 2F,I). Each of these types of clusters may contain cells with intracellular organic bodies. Solitary coccoid cells include examples with rather thick, opaque walls (Fig. 2G) and those with thin translucent walls (Fig. 2H). Again, both of these cell types may possess one or more dark intracellular organic inclusions. Below we detail which types of cells contain REE phosphates and how these relate to the intracellular inclusions.

## Results

REE phosphates occur within a small proportion of microfossil-rich phosphatic (dominantly francolite) nodules from the CHF. Energy dispersive spectroscopy (EDS) and electron diffraction within the transmission electron microscope confirm that both monazite group and xenotime group phosphates are present (Figs S1–5; cf.^12^). Both minerals have rather variable chemistries (Table S1 and Figs S1–5). Monazite contains up to 75 wt% oxides of light REEs (Ce > La > Nd > Pr) plus minor amounts of S (<4%), Ca (<4%) and Sr (<1%). Xenotime contains almost 50% Y, plus up to ~20% of a combination of the heavier REEs (Dy > Gd > Er > Yb > Nd), and minor amounts of Ca (<2%) and U (<1%). More than 90% of all REE phosphate occurring within cells is monazite. There is no obvious zoning within either type of REE phosphate, suggesting rapid precipitation and/or a consistent fluid composition during growth. No detrital igneous or metamorphic monazite or xenotime grains have been observed in these CHF samples.

**Figure 3:**
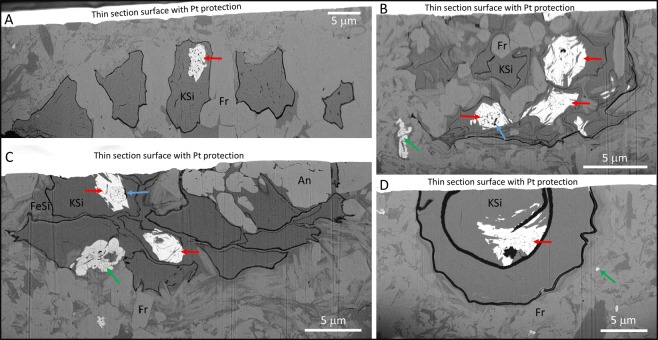
SEM-BSE images of FIB-milled cross sections through selected microfossils illustrated in Fig. 2, highlighting the location of REE phosphate. (**A**) Cross section through the cluster of cells illustrated in Fig. 2A. (**B**) Cross section through the organism illustrated in Fig. 2E. (**C**) Cross section through the cluster of cells illustrated in Fig. 2F. (**D**) Cross section through the organism illustrated in Fig. 2G. Red arrows point to monazite within cells; green arrows to xenotime; blue arrows to best examples of spheroidal organic ICIs; Fr = francolite; KSi = potassium silicates; FeSi = iron/magnesium silicates; An = anatase.

Monazite and xenotime are almost entirely restricted to the interior of specific morphotypes of coccoid cells that form open (Figs 2A–C and 3A) or more closely aggregated (Figs 2D–F and 3B,C) clusters of ten or more cells. One example of monazite preservation in a larger, solitary cell is also demonstrated (Figs 2G and 3D). Cells in open clusters typically contain a single, dark, spherical intracellular inclusion (ICI), similar to that seen in *Glenobotrydion aenigmatis* Schopf 1968. In fact, based on the clustering habit, the size of the ICIs (around 2 µm), and the overall size range of the cells (around 10 µm) many of the open clusters documented here (Figs 2A–C and 3A) are readily accommodated by *G*. *aenigmatis* as originally described by Schopf^3^. Cells in the more closely aggregated clusters typically also have ICIs but are somewhat smaller in diameter and appear to be embedded in diffuse organic matter, which may be bounded by an outer wall (Figs 2D–E and 3B), a feature that is not characteristic of *Glenobotrydion*. We consider these to be cell clusters of uncertain taxonomic affinity. The larger solitary cell (Figs 2G and 3D) appears to be an encysted protist of unknown exact phylogenetic affinity.

The spatial distribution of cell clusters and solitary cells, when viewed in parallel-to-bedding thin sections, does not conform to any obvious geometric pattern. There are no remnants of microbial mats within the studied material, and filamentous mat-building microfossils (empty sheaths or filamentous trichomes) are rare to absent. This condition is similar to the allochthonous nature of organic-rich laminae described previously from the Diabaig Formation^13^, the basal formation of the Torridon Group. The allochthonous nature of scrappy and poorly preserved sheaths (e.g., Fig. 6 of ref.^13^), in combination with the exquisite preservation characterizing the cell clusters described here, suggests that the cells associated with REE phosphates were originally planktonic in nature, but it cannot be ruled out that these deposits have preserved both allochthonous (planktonic) and *in situ* (benthic) micro-organisms. Microbial planktonic blooms, fed by ion/metal enriched riverine input to the lakes^11^, likely sequestered biological phosphate, serving as a source for P_i_ mineralization after settling to the lake bottom sediments.

**Figure 4:**
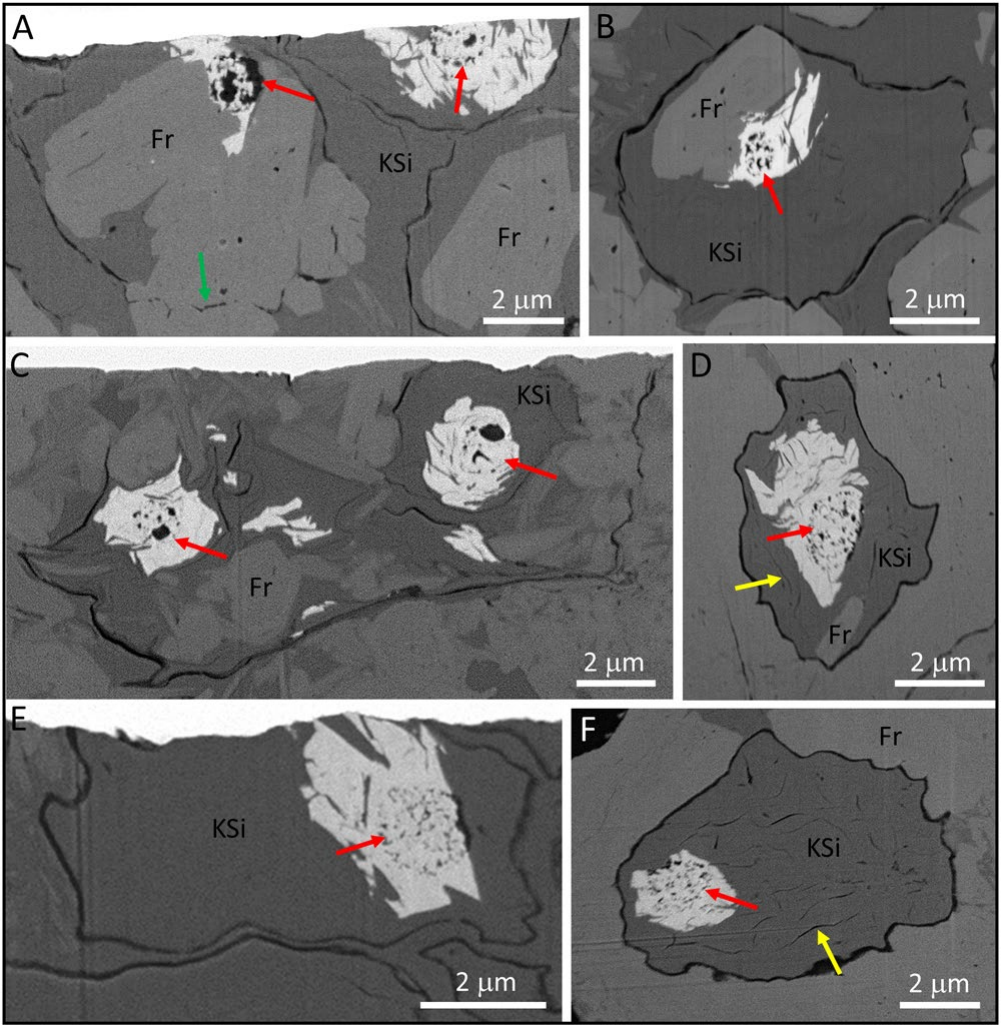
SEM-BSE images of the details of cells containing REE phosphate. (**A**,**B**) Three cells within an open cluster that contain REE phosphate (white) associated with spheroidal organic ICIs (red arrows), plus occasional wisps of black organic material preserved in francolite or clay minerals. Note how large francolite grains disrupt the cell wall in A (green arrow). (**C**) Two cells from the organism illustrated in Fig. 2E that contain multiple organic ICIs (black material) arranged in a roughly spheroidal pattern and associated with monazite (red arrows). (**D**,**F**) Two cells from the cluster illustrated in Fig. 2A with beaded spheroidal masses of black organic material mostly preserved in REE phosphate (red arrows), plus numerous curvi-linear organic microstructures preserved in clay minerals (yellow arrows). (**E**) A cell from the cluster illustrated in Fig. 2F showing a well-defined beaded spheroidal organic microstructure preserved mostly in REE phosphate (red arrow).

**Figure 5:**
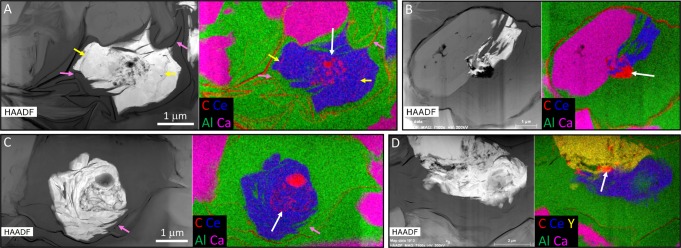
Chemistry of REE phosphates and host cells. (**A**,**C**) Two cells from the organism illustrated in Fig. 2E showing beaded spheroidal organic ICIs (white arrows) entirely preserved in monazite (indicated by blue Ce zone in the composite maps). Note also very delicate wispy organic material preserved within the monazite (yellow arrows in **A**) which also continues in a more flattened state within adjacent clay minerals (pink arrows). Note that remainder of cell is infilled with K-silicate minerals (Al, green), whereas francolite (Ca, pink) only occurs outside of these cells. (**B**) Cell from an open cluster with very distinct carbonaceous ICI (white arrow) partially mineralised by monazite (Ce, blue). (**D**) Cell from the cluster illustrated in Fig. 2B with indistinct carbonaceous ICI (white arrow) associated with both monazite (Ce, blue) and xenotime (Y, yellow). HAADF panels are high angle annular dark-field scanning transmission electron microscopy images in which low mass material (e.g., carbon) shows up black. The elemental maps were obtained using *ChemiSTEM* energy dispersive spectroscopy (EDS). Full sets of elemental maps for these cells are given in Figs S1–4 and EDS spectra in Fig. S5.

REE phosphates typically occur in only 10–50% of the cells within a cluster, with clay minerals and francolite the more common fossilizing phases. In cells that contain REE phosphates, the remainder of the cell is usually mineralized by K-rich clays (Figs 3–5 and S1–4) but francolite can sometimes occur (Figs 4A,B,D and 5B; Fig. S2); Mg-Fe-rich clays are restricted to the immediate vicinity of some cell walls (Figs 3C and S1–4). Almost all of the cells containing REE phosphate also possess well preserved organic ICIs. The preservation of ICIs varies within cell clusters, but in light microscopy there appear to be two general types: (1) dense, rounded blebs (“spots”) that appear to be more or less solid carbonaceous material (Fig. 2A,C–E), and, (2) broadly spherical clusters of carbonaceous beads (Fig. 2C, arrows). Electron microscopy examination shows a more heterogeneous nature to the ICIs in general, as most ICIs appear to contain an admixture of smaller and larger organic particles (Figs 4A,C and 5). However, the broadly spherical nature of the beaded forms is quite distinct in Figs 3C and 4A,D, E, where the undistorted circular shape of these inclusions is retained perfectly embedded within the REE phosphate. Very delicate, wispy organic microstructures are also occasionally preserved within the REE phosphate (Fig. 5A, yellow arrows) which are most logically interpreted as the remains of a cell membrane or inner wall layer that has detached from the cell wall during fossilization. In contrast, authigenic clay mineral growth in these same cells appears to have acted as a physical front that pushed organic material to platelet boundaries, forming wispy, curvi-linear organic structures (Fig. 4D,F, yellow arrows). Larger crystals of francolite tend to cause distortion of original cell components, which manifest themselves as crenulated and angular portions of thicker cell walls; this is particularly evident in Figs 3A and 4A.

REE phosphates also occur in larger (~20μm diameter) thick-walled vesicles that enclose a thick-walled cell, which in turn contains a single dense ICI (Figs 2G and 3D), though this has only been observed on a single occasion. Here the REE phosphate appears to have grown within a thick, inner (cyst?) wall subsequently encasing a single granular solid ICI. REE phosphates have not yet been observed in simple coccoid unicells with thin walls (e.g., Fig. 2H), nor in clusters of coccoids where none of those cells contain organic inclusions (e.g., Fig. 2I). Only very rarely are REE phosphates observed outside of cells, where they occur as isolated nano-grains (Fig. 3D). These observations point to the likelihood that REEs were originally sequestered within certain cells as a result of biological activity, and that they did not arrive exogenously in association with later interstitial fluid flow.

## Discussion

At about 1000–900 Ma, the Grenville Orogen was a major geological structure located to the South-West of the Torridon lakes^14^. Paleocurrent and basin analysis data from the CHF strongly suggests Cailleach Head lakes were supplied by large rivers with heavy sediment loads, part of a large-scale trunk system flowing parallel to the Grenville Orogen front and transporting away material eroded in the orogen^7,8,15^. Detrital zircon and rutile ages and provenance studies provide constraints on the different magmatic and metamorphic units in these mountains which could have acted as sediment/REE sources. These include the 1650 Ma Trans-Labrador batholith^15^ and metamorphic rocks associated with the 1900-1700 Ma Makkovik-Ketilidian-Rhinnian (MKR) Orogen that were both uplifted and eroded during the Grenvillian episode^9^. Studies of modern environments show that large rivers can contain c. 10–1000 times the concentration of dissolved REEs compared to seawater^16,17^.

The location of almost all of the REE phosphates in the central portions of fossilized cells, along with their spatial association with ICIs, strongly suggests that both REEs and phosphate were actively transported and sequestered internally by the living cells. Both eukaryotic algae and cyanobacteria today possess mechanisms for the biological uptake and sequestration of REEs^18–20^ and phosphate^21,22^ in the form of polyphosphate granules. In lacustrine ecosystems, photosynthetic microalgae retain the highest levels of REEs within the trophic web^23^. The incorporation of heavy metals into intracellular polyphosphate^24–26^ is one of several strategies for metal detoxification that occurs in extant microalgae^27^. Given the strong affinity of REEs for the phosphate ligand^28^, REEs may have been preferentially concentrated into such granules over other metals such as iron and copper. Hence, polyphosphate granules, which are quite widespread in unicellular organisms today^29^, could have been a ready intracellular source of both REEs and P_i_ during early phases of authigenic mineralization in the CHF.

When degrading polyphosphate granules were swept up into the ICIs as part of the plasmolyzed cell contents, REE phosphate could rapidly precipitate, resulting in the observed association of REE phosphate with spheroidal masses of organic material. The presence of significant sulfur in some of the REE phosphate grains (Table S1) also supports rapid precipitation, before sulfate was exhausted by anaerobic sulfate reduction (cf.^1^). The contrasting distribution of francolite in most of these cells (i.e. dominantly exterior to cell walls), plus the spatial separation of REE-phosphate from francolite by intervening clay minerals, points to a different source for the Ca (and P) for francolite. This could have been from circulating porewaters and/or locally made available during the anaerobic bacterial decomposition of EPS (cf.^30^).

Reports of cell contents preserved as ICIs within Precambrian fossilized cells are relatively uncommon (e.g.^3,31^). The observation of cellular inclusions in Precambrian microfossils, began a debate when so-called “spot cells” (originally “eye spots” in ref.^32^) from the Bitter Springs Formation were interpreted as fossilized putative nuclei^2^, or, in some cases, pyrenoids^33^. These initial inferences were later re-interpreted as degraded cell contents by Knoll & Barghoorn^34^ and followed up by studies that compared degraded cultures directly with fossils^35^ and artificially-silicified cultures^36^. Francis *et al*. (ref.^36^, Fig. 17) were even able to reproduce putative nuclei (“spot cells”) in moribund cultures of the cyanobacterium, *Chlorogloea* sp. demonstrating conclusively that degrading prokaryotic cells were capable of producing ICIs that superficially resemble possible eukaryotic nuclei. These earlier studies on the origin of ICIs, focussed exclusively on preservation in chert, and found no conclusive evidence that any Precambrian microfossils harboured preserved nuclei.

With the discovery and description of ICI’s found preserved in cells in the Doushantuo phosphorite e.g.^37^, interest has now shifted to phosphatization as a preservational mechanism. Subcellular preservation in fossils from the Doushantuo and the Torridonian is different with respect to size of fossils and mineralogy. Proposed nuclei from the Doushantuo are large (>50 µm in diameter), and they occur in very large (>150 µm) cells^37^. Doushantuo ICIs are substantially larger than the entire cells examined in this report. This size difference is also associated with very different forms of mineralization – including secondary mineral textures and void fillings – that are quite unlike those seen in the Torridonian. Specifically, preservation associated with potential cell membranes and nuclei are characterized by botryoidal aggregates and late-stage overgrowths associated with secondary phosphatization^38^. In general, authigenetic mineralization seen here in the Torridonian is associated with cryptogranular textures that have not been subjected to recrystallization^6^. Overall ICIs found preserved in the Torridon phosphates appear to be taphonomic features formed by the condensation or degradation of the entire contents of a given cell. Sub-cellular structures in living cells include membrane-bound organelles (mainly plastids or nuclei), macromolecular assemblies (such as chromosomes, ribosomes, or microtubule-based assemblies such as centrioles and basal bodies), or cytoplasmic granules (e.g. glycogen granules, various starch bodies, pyrenoids, or polyphosphate granules). Of these, it seems logical to us that cytoplasmic granules have the greatest potential for preservation (in agreement with other recent work^39^), followed by membrane-bound organelles, with unbounded macromolecular assemblies the least likely to remain structurally intact during fossilization. The extent to which any such sub-cellular structures are preserved in any Precambrian cells continues to be a matter of debate^31^ and we are making no specific claims here.

We interpret the ICIs preserved in the CHF as largely plasmolyzed cell membranes and/or permeable wall layers which themselves contain the degraded remains of entire cells. The shape of both dense organic spots and clusters of smaller bead-like forms is always roughly spherical, consistent with plasmolysis. In fact, it seems somewhat remarkable that the sphericity of the ICIs is so well retained, especially given the clearly crystalline form of the encasing REE phosphate (e.g. Fig. 4E). Nonetheless, there are clear exceptions to a simple plasmolysis model, as electron microscopy reveals that some spheroidal ICIs preserved in REE phosphate are not the only organic inclusion within the host cell. For example, there are sometimes very delicate wispy organic features preserved in REE-phosphate (Fig. 5A, yellow arrows), that also extend into clay minerals as single curvi-linear organic structures (Fig. 5A,C, pink arrows); these are interpreted as part of a cell membrane or inner cell wall layer that disassociated with the rest of the cell contents during plasmolysis. There are also organic blebs and multiple wispy, membranous features that persist in the space between outer cell walls and some inner spheroidal ICIs (Figs 3C and 4B–D). Thus some preserved organic features appear to be the taphonomically-altered components of original cytoplasm. In these cases it is possible that the spheroidal ICIs are remnants of either cytoplasmic inclusions (storage granules) or perhaps even membrane-enclosed plastids. We also commonly observe relatively thick, and sometimes multilayered, organic walls enclosing multiple cells (Figs 3B and 4C) that are perhaps best interpreted as mucilaginous sheaths or EPS. These combined observations indicate greater complexity than that seen in taphonomic decay experiments using simple prokaryotes^34^ but certainly do not rule out the possibility of prokaryote affinity for most of the REE-hosting cells.

Notwithstanding their phylogenetic origin, the fidelity of preservation of these 1 billion-year-old cell contents is remarkable. Examples of Ca-phosphate (e.g., francolite) providing high fidelity, three-dimensional fossil preservation are numerous, for example the much-lauded Doushantuo biota^4^. REE phosphates may have the potential to take this fidelity even further, especially because they appear to precipitate rapidly and early on in the decay process. With the application of higher spatial resolution and higher sensitivity analytical techniques to the Torridon biota and other phosphatised biotas^37–40^ we are reaching a new threshold in the study of Precambrian microorganisms, whereby the taphonomy of intracellular structure can now be addressed directly. This has the potential to provide detailed insight to the biology and environmental chemistry of ancient ecosystems.

## Methods

Samples of phosphatic nodules were collected during fieldwork in 2011–2013. Polished geological thin sections of various thicknesses (c. 25 μm up to c. 100 μm) were prepared at Oxford University and subsequently at The University of Western Australia (UWA). Thin sections were examined at UWA in transmitted and reflected light using *Leica DM2500M* and *Zeiss Axioskop* microscopes, and images were captured using a digital camera and *ToupView* imaging software.

TEM wafers were prepared using a dual-beam FIB/SEM system (*FEI Helios NanoLab G3 CX*) at the Centre for Microscopy, Characterisation and Analysis (CMCA), UWA. Electron beam imaging was used to identify specimens of interest (previously located using transmitted and reflected light microscopy) in the geological thin sections coated with c. 10 nm of gold, allowing site-specific TEM wafers to be prepared. In order to gain a full understanding of the location of the REE phosphate in relation to the cells we performed 3D SEM imaging in the lead up to wafer extraction. This involved covering the surface of entire cell clusters with protective ~1 μm thick Pt pads and milling c. 20 μm deep trenches around these features using a 30 kV and 21 nA Ga^+^ beam. We then milled (using a 30 kV and 9.3 nA Ga^+^ beam) from opposite directions towards the centres of the cell clusters. During milling, SEM-BSE images were captured to show the morphology of the cells and phosphate minerals, and at selected points EDS elemental maps and spectra were acquired to demonstrate the chemistry of the microstructures.

During the above procedure specific REE phosphate grains were targeted for wafer extraction and these sub-areas were thinned to around 1.5–2 μm using a 30 kV and 2.5 nA Ga^+^ beam. These wafers were extracted from the geological thin section using an *in-situ EasyLift EX Nano-Manipulator* and welded to an *Omniprobe®* copper TEM holder using platinum connector strips. Final thinning of the wafers to c.100–150 nm was performed *in situ* on the holder using a series of low ion beam currents (0.79 nA then 0.23 nA), before a final cleaning stage was performed (5 kV and 41pA Ga^+^ beam). Unlike traditional TEM grids where samples are deposited on carbon film, this welding protocol means that there is no carbon film underneath the wafer, simplifying subsequent carbon elemental mapping in the TEM. Any risks of surface contamination leading to cell-like artifacts (for example during thin section preparation and polishing) are here mitigated because FIB preparation of TEM sections allows features lying mostly or entirely below the surface of a thin section to be targeted. There is no void space or fractures within the phosphate nodules and no glue/resin from thin section preparation was encountered at any time during the analyses.

TEM data were obtained using a *FEI Titan G2 80–200* TEM/STEM with *ChemiSTEM Technology* operating at 200 kV, located in CMCA at UWA. Data obtained included bright-field TEM images, HAADF (high angle annular dark-field) STEM images, EDS (*ChemiSTEM*) elemental maps, EDS spectra at a variety of energy ranges, and selected area electron diffraction patterns. Quantification of EDS spectra, in order to obtain approximate mineral compositions for monazite and xenotime, was performed using a standardless Cliff-Lorimer method via the *Bruker Esprit* software following suitable background removal.

## Supplementary information

Supplementary Information

## Data Availability

All data and materials are freely available within supplementary information or on request from DW.
