# Supplementary material for: 1 billion-year-old cell contents preserved in monazite and xenotime
Source: Sci Rep. 2019 Jun 21;9:9068. doi: 10.1038/s41598-019-45575-4 (PMC6588638; doi:10.1038/s41598-019-45575-4)
Supplement: Supplementary file 1 — Supplementary Information [file 41598_2019_45575_MOESM1_ESM.pdf]

Supplementary Information for

1 billion-year-old cell contents preserved in monazite and xenotime

David Wacey, Eva Sirantoine, Martin Saunders, and Paul Strother

Corresponding author: David Wacey
Email: David.Wacey@uwa.edu.au

This PDF file includes:

Figs. S1 to S5
Table S1

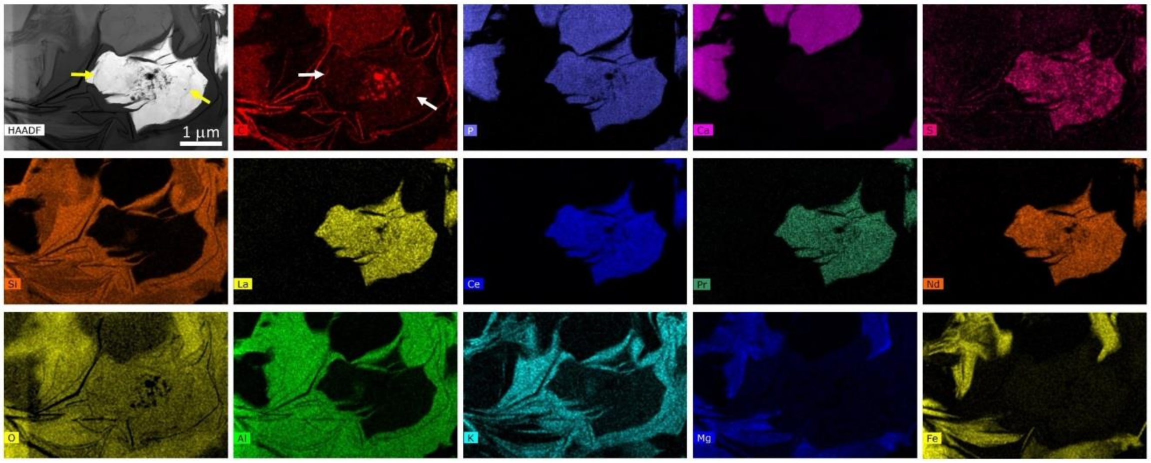

Fig. S1. Full set of scanning transmission electron microscopy – energy dispersive spectroscopy (STEM-EDS) elemental maps for the cell and REE phosphate shown in Figure 5A of the main article. Note the presence of the REEs La, Ce, Pr and Nd, as well as S in the phosphate inside the cell. The remainder of the cell is filled with K-rich clay minerals, whereas calcium phosphate (francolite) and Mg-Fe clay minerals are found outside of the cell and/or in the immediate vicinity of the cell walls. Note also the delicate wispy organic structure preserved in REE phosphate (arrows in HAADF image and C map), here interpreted as either the cell membrane or an inner layer of the cell wall.

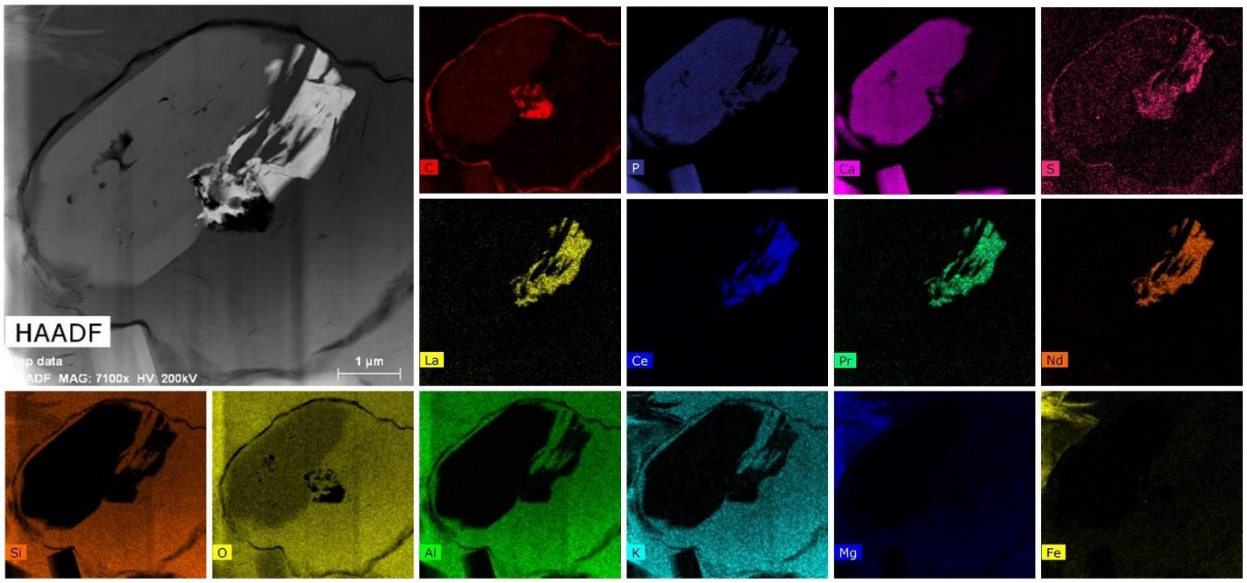

Fig. S2. Full set of STEM-EDS elemental maps for the cell and REE phosphate shown in Figure 5B of the main article. Note the presence of the REEs La, Ce, Pr and Nd, as well as S in the phosphate inside the cell. The remainder of the cell is filled with K-rich clay minerals and calcium phosphate (francolite). Mg-Fe clay minerals are only found outside of the cell.

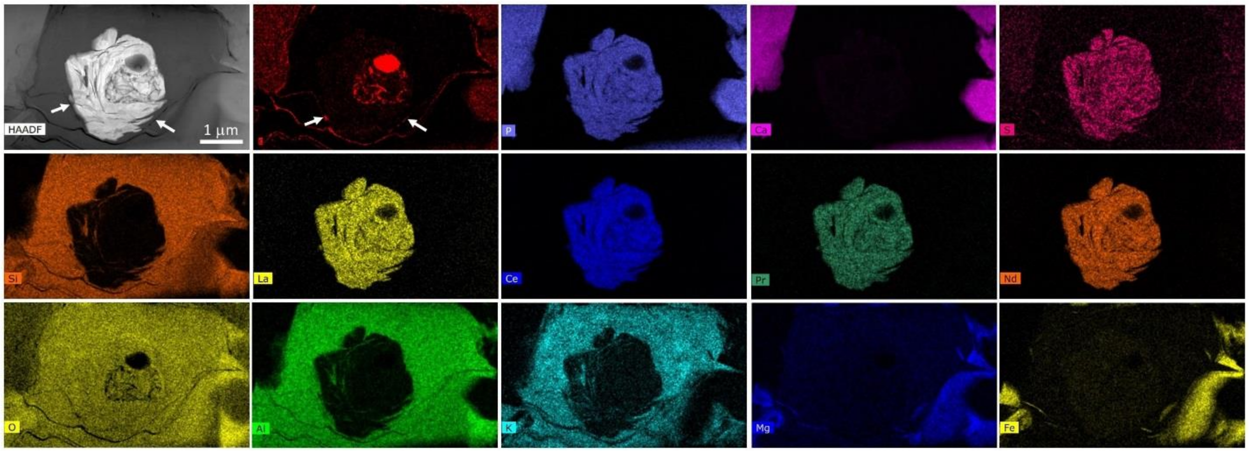

Fig. S3. Full set of STEM-EDS elemental maps for the cell and REE phosphate shown in Figure 5C of the main article. Note the presence of the REEs La, Ce, Pr and Nd, as well as S in the phosphate inside the cell. The remainder of the cell is filled with K-rich clay minerals, whereas calcium phosphate (francolite) and Mg-Fe clay minerals are found outside of the cell and/or in the immediate vicinity of the cell walls. Note also the delicate organic structure that appears to have pulled away from the cell wall (arrows in HAADF image and C map), here interpreted as either an inner layer of the wall or the cell membrane.

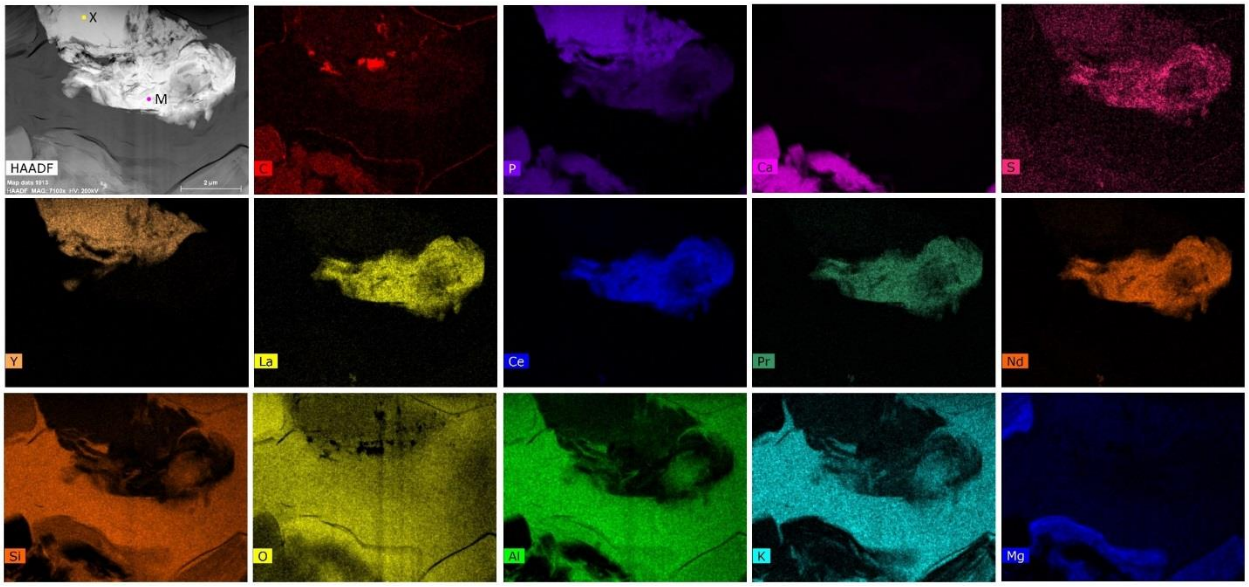

Fig. S4. Full set of STEM-EDS elemental maps for the cell and REE phosphate shown in Figure 5D of the main article. Note the presence of the light REEs La, Ce, Pr and Nd, as well as significant S in the lower portion of the phosphate inside the cell, whereas the upper portion is Y-rich. The heavier REEs Gd, Dy, Er and Yb also occur in this Y-rich phosphate (see Fig. S5) but peak overlaps and background signal prevent clean elemental maps of these being produced. The remainder of the cell is once again filled with K-rich clay minerals, whereas calcium phosphate (francolite) and Mg-Fe clay minerals are found outside of the cell and/or in the immediate vicinity of the cell walls.

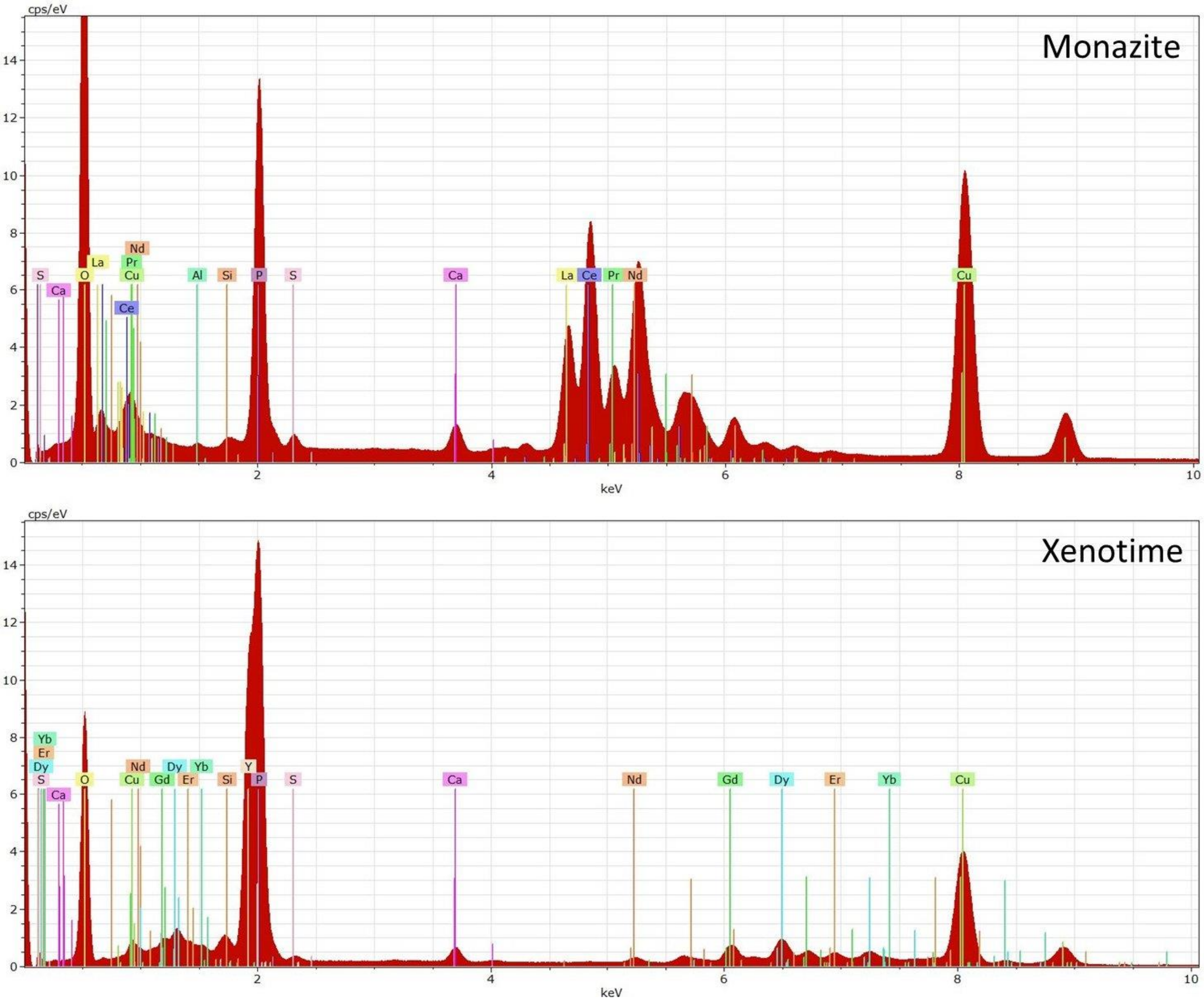

Fig. S5. TEM-EDS spectra for our monazite group and xenotime group REE phosphates (from areas marked M and X respectively in Fig. S4). Note that Cu is from the Cu grid to which the TEM wafer is attached, and the small Al and Si signals are inferred to be from the surrounding clay minerals (although it is also plausible that they are present in trace amounts in the phosphates).

Table S1. Quantitative mineral analysis of the REE phosphates (M1-M5 = monazite group; X1-X5 = xenotime group) found within the CHF cells. Note that this is a standardless quantification based on the Cliff-Lorimer method using STEM-EDS data; '–' = below detection limits.

	**M1**	**M2**	**M3**	**M4**	**M5**	**X1**	**X2**	**X3**	**X4**	**X5**
**P_2O_5**	28.8	24.5	23.9	23.7	27.2	33.9	33.3	29.8	35.4	33.9
**SO_3**	3.7	2.4	3.6	0.7	3.9	-	-	-	-	-
**CaO**	4.1	3.1	3.5	1.7	3.8	1.5	1.2	1.2	1.4	0.8
**SrO**	0.7	0.6	0.7	0.3	0.7	-	-	-	-	-
**Y_2O_3**	-	-	-	-	-	43.6	44.8	41.4	46.1	46.0
**La_2O_3**	10.9	21.4	16.3	21.8	11.5	-	-	-	-	-
**Ce_2O_3**	35.4	35.1	34.6	41.1	33.7	-	-	-	-	-
**Pr_2O_3**	-	-	4.5	1.2	3.9	-	-	-	-	-
**Nd_2O_3**	15.5	14.4	16.7	12.3	14.4	1.7	1.5	3.7	1.7	-
**Gd_2O_3**	-	-	-	-	-	7.2	6.7	6.5	5.2	1.7
**Dy_2O_3**	-	-	-	-	-	9.5	9.5	8.1	7.2	10.6
**Er_2O_3**	-	-	-	-	-	4.1	4.6	5.9	1.7	3.5
**Yb_2O_3**	-	-	-	-	-	1.6	1.9	6.7	0.8	2.7
**UO_2**	-	-	-	-	-	0.5	0.6	0.3	0.7	-
**Total (%)**	**99.1**	**101.5**	**103.8**	**102.8**	**99.1**	**103.6**	**104.1**	**103.6**	**100.2**	**99.2**
